# Modeling tumor development and metastasis using paired organoids derived from patients with colorectal cancer liver metastases

**DOI:** 10.1186/s13045-020-00957-4

**Published:** 2020-09-03

**Authors:** He Li, Weixing Dai, Xi Xia, Renjie Wang, Jing Zhao, Lingyu Han, Shaobo Mo, Wenqiang Xiang, Lin Du, Guangya Zhu, Jingjing Xie, Jun Yu, Nan Liu, Mingzhu Huang, Jidong Zhu, Guoxiang Cai

**Affiliations:** 1 Interdisciplinary Research Center on Biology and Chemistry, Shanghai Institute of Organic Chemistry, Chinese Academy of Sciences, Shanghai, China; 2 University of the Chinese Academy of Sciences, Beijing, China; 3 Department of Colorectal Surgery, Fudan University Shanghai Cancer Center, Shanghai, China; 4 Department of Oncology, Shanghai Medical College, Fudan University, Shanghai, China; 5 Department of Surgery, Johns Hopkins University School of Medicine, Baltimore, MD USA; 6 Department of Medical Oncology, Fudan University Shanghai Cancer Center, Shanghai, China; 7 Center for Excellence in Molecular Synthesis, Shanghai Institute of Organic Chemistry, Chinese Academy of Sciences, Shanghai, China

**Keywords:** Colorectal cancer, Tumor metastasis, Preclinical model, Paired organoids, SOX2

## Abstract

Tumor metastasis accounts for the majority of cancer-related deaths; it is therefore important to develop preclinical models that faithfully recapitulate disease progression. Here, we generated paired organoids derived from primary tumors and matched liver metastases in the same colorectal cancer (CRC) patients. Despite the fact that paired organoids exhibit comparable gene expression and cell morphology, organoids from metastatic lesions demonstrate more aggressive phenotypes, tumorigenesis, and metastatic capacity than those from primary lesions. Transcriptional analyses of the paired organoids reveal signature genes and pathways altered during the progression of CRC, including SOX2. Further study shows that inducible knockdown of SOX2 attenuated invasion, proliferation, and liver metastasis outgrowth. Taken together, we use patient-derived paired primary and metastatic cancer organoids to model CRC metastasis and illustrate that SOX2 is associated with CRC progression and may serve as a potential prognostic biomarker and therapeutic target of CRC.

To the Editor,

Tumor heterogeneity plays a key role in cancer progression and therapy resistance [1]. However, knowledge of how tumor heterogeneity arises and contributes to disease progression is still limited [2]. Recent advances in organoid culture have been successfully established in a variety of solid tumors [3–5]. Tumor organoids retain the histological complexity and genetic heterogeneity of parental tumors, even after many passages [6], providing a wide range of applications for cancer research. Organoids have enormous potential for the identification of optimal treatment strategies in individual patients [6]. For example, human CRC organoids derived from primary tumors [5] and liver metastases [7] have been reported as precision medical models for assessing drug responses. However, paired organoids have not been studied as a model for CRC progression. In the present study, we used paired organoids derived from primary and liver metastatic tumors of CRC patients to model cancer metastasis. Through in vitro and in vivo studies and transcriptional analyses of the paired organoids, we revealed key genes associated with CRC liver metastasis, which could be translated into therapeutic targets or prognostic biomarkers for disease treatment. A total of 24 organoids have been established (Table S1). The library contained 2-paired organoid lines from patients P13 and P21. Particularly, P13 carried two primary tumors. The 13a and 13b organoids were established from the primary tumor, while 13L organoid was established from a synchronous liver metastatic tumor. Organoids of 21a and 21L were established from primary tumor and synchronous liver metastasis of the patient P21, which data demonstrated in Additional files (Supplementary Table S1 and Fig. S1, S2, S4, and S5). Histopathological structures and the intestinal epithelial marker CDX2 of parental tumor were well preserved in organoids (Fig. S1).

Invasion is a fundamental step in tumor progression toward metastasis. To study collective invasion, we cultured paired organoids in a 3D invasion matrix (Fig. 1a). Although we did not observe collective protrusive migration in organoids derived from primary lesions, metastatic organoids exhibited robust protrusive migration into 3D invasion matrix (Fig. 1b and Fig. S2A and B). Besides, the expression level of MMP-2 (matrix metalloproteinase 2) and Ki67 was significantly higher in metastatic organoids than that in the primary organoids (Fig. S2C-E). In subcutaneous xenotransplantation of paired organoids (Fig. 1c), the growth rate and volume of 13L organoids derived xenograft tumors was significantly higher than that of 13a and 13b organoids derived tumors (Fig. 1d and e). Furthermore, we successfully generated organoids from xenografts, histology, and Ki-67 expression analysis of xenografts, as well as organoids derived from these xenografts, demonstrated similarity to the original parental tumors (Fig. 1f and Fig. S2F). We next performed splenic injection of the paired organoids to assess the development of liver metastases (Fig. 1g). The 13L organoids formed macrometastatic tumors in the livers (Fig. 1h and i), whereas 13b organoids and 13a organoids failed to colonize and had a negative expression of Ki67 in the liver (Fig. S2G).

**Fig. 1:**
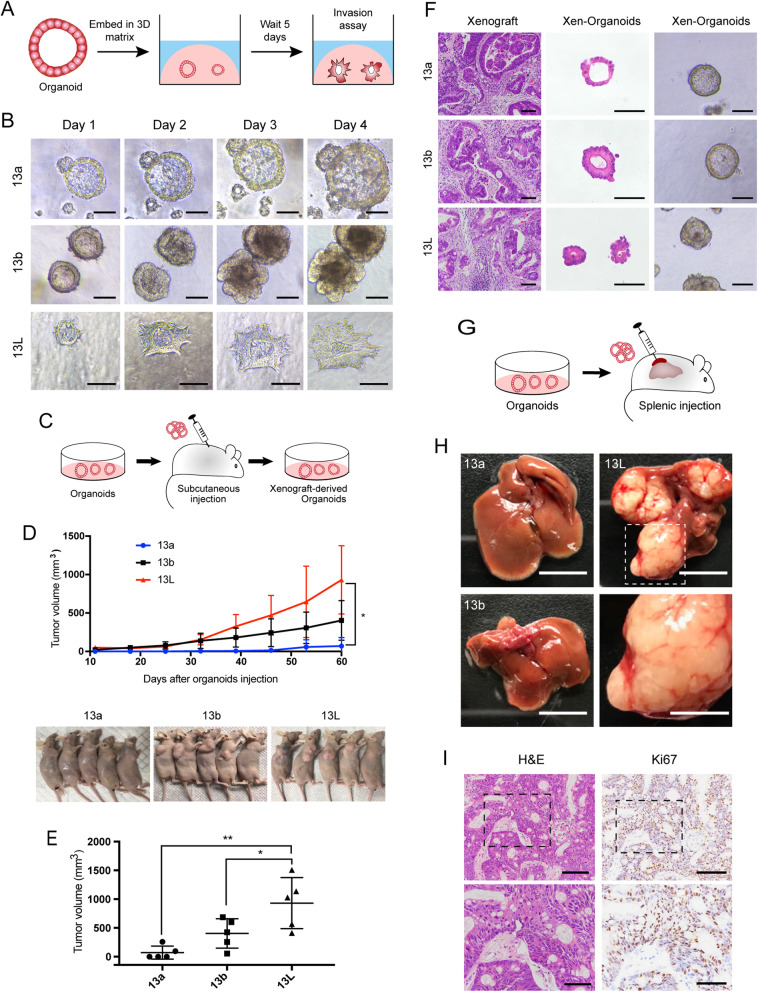
Patient-derived paired organoids provide invasion and transplantable models for human CRC progression. **a** Schematic of 3D invasion assay using paired organoids. **b** Representative micrographs of organoids in 3D invasion assay. Tumor organoids showed the smooth and protrusive leading fronts, respectively. The scale bar represents 100 μm. **c** Schematic of the subcutaneous organoid injection. **d** Organoids were injected subcutaneously into the flank region of nude mice for 60 days. Tumor volumes were monitored over time (top). *n* = 5 mice per group. Error bars indicate SEMs. *p = 0.0146 (one-way ANOVA). **e** Tumor volume of the mice at day 60 after inoculation. Each dot indicates individual mice. *n* = 5 mice per group. Error bars indicate SEMs. *p = 0.0433, **p = 0.002 (one-way ANOVA). **f** Representative bright-field images of organoids together with H&E staining of xenografts generated from organoids, and organoids derived from the xenografts. The scale bar represents 100 μm. **g** Schematic of the hepatic metastasis assay by splenic organoid injection. **h** Representative macroscopic photographs of the whole liver. *n* = 5 mice per group. Top, scale bar of the whole liver represents 1 cm. Bottom, high magnification of inset. Scale bar, 5 mm. **i** Representative histopathology and Ki67 staining of liver metastatic lesions generated from splenic injection of 13L organoids. Top, the scale bar represents 100 μm; bottom, the scale bar represents 50 μm

We then performed gene expression analysis in the paired organoids and tumor tissue from patient 13. There were 33 genes (*P* < 0.05; fold change > 2.5) that were significantly upregulated in metastatic organoids (Fig. 2a and Fig. S3A and B), including the transcription factor SOX2. Previous studies have shown that SOX2 plays critical roles in embryonic pluripotent stem cells [8] and that SOX2 is abnormally expressed in many types of cancer [9–12]. The differential expression of SOX2 in paired organoids was consistent with the RNA-seq data (Fig. 2b and c), and SOX2 was also highly expressed in the metastatic tissues (Fig. 2d), while relatively low expression in normal colon tissues (Fig. S3C-F). SOX2 is also highly expressed in metastatic organoids and tissues of the other paired organoids (Fig. S4A and B).

**Fig. 2:**
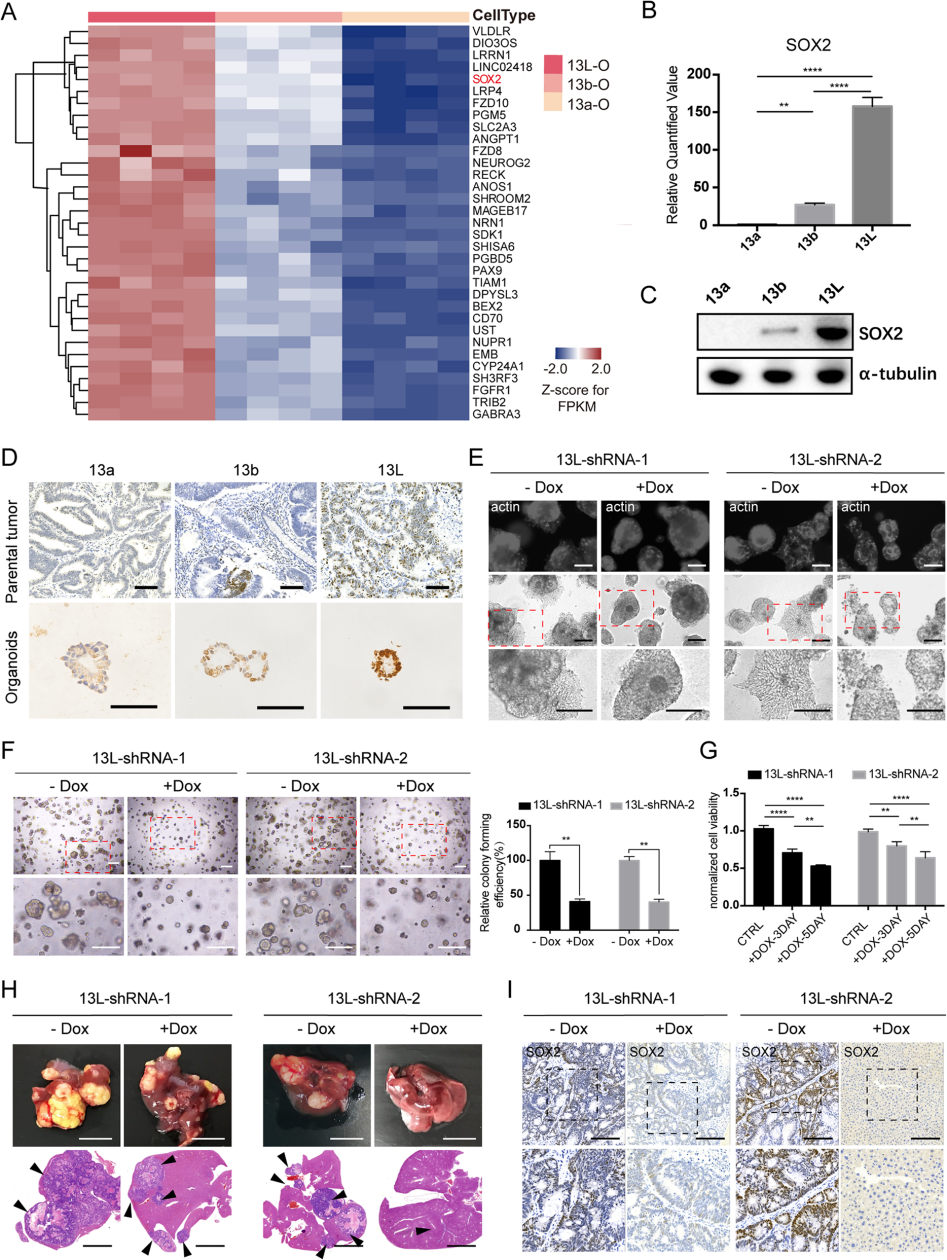
SOX2 plays an important role in colorectal metastasis. **a** Hierarchical clustering heatmaps of the 33 significantly upregulated genes in 13L organoids. **b** qRT-PCR analysis of SOX2 in paired organoid lines. Values were normalized to mean levels in 13a organoids. Error bars indicate SEMs. **p = 0.0050, ****p < 0.0001 (one-way ANOVA). **c** Western blot analysis of the SOX2 protein expression in paired organoid lines. α-tubulin was used as a loading control. **d** Representative IHC sections for SOX2 in human colorectal tumor tissues and organoids. The scale bar represents 100 μm. **e** Representative micrographs of Dox-transduced the 13 L-shRNA-1/2 organoids after 5 days and stained with phalloidin–F-actin. The scale bar represents 100 μm. **f** Representative micrographs of colonies arising from the 13L-shRNA-1/2 organoids (top), with magnified insets showing colonies (bottom). The scale bar represents 200 μm. Colony forming efficiency in **f** was calculated and compared (right). Error bars indicate SEMs. **p = 0.0078, (two-way ANOVA). **g** Proliferation of the 13L-shRNA-1/2 organoids were examined by CTG cell viability assays following 3- or 5-days growth in the presence or absence of Dox. Error bars indicate SEMs. **p = 0.0019, ****p < 0.0001 (two-way ANOVA). **h** Representative macroscopic photographs (top) and histology sections (H&E, bottom) of the livers of NOD mice transplanted with the 13L-shRNA-1/2 organoids. Arrowheads, metastatic foci. *n* = 5 mice per group. Scale bars, 1 cm (top) and 4 mm (bottom). **i** Representative SOX2 immunostaining of liver metastases of the 13L-shRNA-1/2 organoids. Scale bars, 200 μm (top) and 100 μm (bottom)

To investigate the role of SOX2 in CRC progression, doxycycline (Dox) inducible expression of shRNA targeting SOX2 was established in metastatic organoids (Fig. S4C and D). SOX2^−^ organoids exhibited the reduced ability of invasion, colony-forming efficiency, and cell viability in metastatic organoid lines (Fig. 2e-g and Fig. S4E-G). Furthermore, the metastatic organoids efficiently formed large metastatic tumors in control groups (Dox untreated), whereas the SOX2^−^ organoids showed no or few engraftments (Fig. 2h). The downregulation of SOX2 and Ki67 was further confirmed by immunohistochemistry (Fig. 2i and Fig. S4H). We then overexpressed SOX2 in primary organoids and found that organoids with overexpressed SOX2 exhibited increased ability of invasion and proliferation when compared with control organoids (Fig. S5). Taken together, these findings demonstrate that SOX2 expression is sufficient and necessary for CRC organoids to exhibit the metastatic potential.

In summary, the present study highlights the potential of patient-derived paired primary and metastatic cancer organoids as an experimental model for investigating CRC progression. We identified a significantly dysregulated gene between paired organoids, SOX2, which could be a prognostic biomarker, and perhaps a potent therapeutic target in the treatment of CRC.

## Supplementary information

**Additional file 1: Supplementary Table S1.** Summary of patient-derived CRC organoid lines and corresponding clinical data.

**Additional file 2: Supplementary Figures. Figure S1.** Paired organoids derived from primary and metastatic CRC recapitulate the histopathological structure of parental tumor. **A** and **B**, Organoids architecture resembles parental tumor epithelium. Representative bright-field images of organoids together with H&E staining of parental tumors and patient-derived organoids. The scale bar represents 100 μm. **C** and **D**, Representative IHC sections for the intestinal epithelial marker CDX2. The scale bar represents 100 μm. **Figure S2.** Organoids derived from liver metastatic lesions exhibited the most aggressive phenotypes with significant high tumorigenic and metastatic potential. **A**, Representative micrographs of organoids in 3D invasion assay. Tumor organoids showed the smooth and protrusive leading fronts, respectively. **B**, Micrographs of the paired organoids stained with phalloidin–F-actin (right). The scale bar represents 100 μm. **C**, qRT-PCR analysis of MMP-2 in paired organoid lines. Error bars indicate SEMs. *P=0.0207, **P =0.0083, ***P=0.0008 (one-way ANOVA; left), ***P=0.0006 (Unpaired t test; right). **D**, Western blot analysis of the MMP-2 protein expression in paired organoid lines. α-tubulin was used as a loading control. **E**, Representative IHC sections for Ki67 in human colorectal tumor tissues and paired organoid lines. The scale bar represents 100 μm. **F**, Representative IHC sections for Ki67 in organoid xenografts and organoids derived from xenografts. The scale bar represents 100 μm. **G**, Representative gross image, histopathology and Ki67 staining of whole liver from primary organoid xenografts. The scale bar of the whole liver represents 1cm. The black scale bar represents 100 μm. **Figure S3. A**, Representation of the up-regulated genes in 13L organoids. Error bars indicate SEMs. *P = 0.0283, ***P = 0.0004, ****P < 0.0001 (one-way ANOVA). **B**, qRT-PCR analysis of the up-regulated genes in 13L organoids. Values were normalized to mean levels in 13a organoids. Error bars indicate SEMs. *P=0.02, ***P=0.0008 ****P < 0.0001 (one-way ANOVA). **C**, RNA sequencing analysis of SOX2 in human colorectal cancer tissues and normal colon tissues. **D,** qRT-PCR analysis of SOX2 in tumor tissues and paired normal colon tissues. Values were normalized to mean levels in normal colon tissues. Error bars indicate SEMs. **P = 0.0018, ****P < 0.0001 (one-way ANOVA). **E**, Western blot analysis of the SOX2 protein expression in tumor tissues and paired normal colon tissues. α-tubulin was used as a loading control. **F**, Representative IHC sections for SOX2 in human colorectal tumor tissues and paired normal colon tissues. The scale bar represents 100 μm. **Figure S4.** Silencing SOX2 in metastatic organoids attenuated cell invasion and proliferation and suppressed liver metastasis in vivo. **A**, qRT-PCR analysis of SOX2 in paired organoid lines (left). Values were normalized to mean levels in 21a organoids. Error bars indicate SEMs. ****P < 0.0001(Unpaired t test). Western blot analysis of the SOX2 protein expression in paired organoid lines. α-tubulin was used as a loading control (right). **B**, Representative IHC sections for SOX2 in human colorectal tumor tissues and organoids. The scale bar represents 100 μm. **C**, qRT-PCR analysis of SOX2 in the 13L-shRNA-1/2 (left) and 21L-shRNA-1/2 (right) organoids after 3 days of Dox-induction. The level of SOX2 was compared to that in the untreated sample. Error bars indicate SEMs. ***P=0.0001, ****P < 0.0001 (two-way ANOVA). **D**, Western blot analysis of the expression of SOX2 in the 13L-shRNA-1/2 and 21L-shRNA-1/2 organoids after 3 days of Dox-induction. α-tubulin was used as a loading control. **E**, Representative micrographs of Dox-transduced the 21L-shRNA-1/2 organoids after 5 days and stained with phalloidin–F-actin. The scale bar represents 100 μm. **F**, Proliferation of the 21L-shRNA-1/2 organoids were examined by CTG cell viability assays following 3- or 5-days growth in the presence or absence of Dox. Error bars indicate SEMs. **p=0.0019, ****P < 0.0001 (two-way ANOVA). **G**, Representative micrographs of colonies arising from the 21L-shRNA-1/2 organoids (top), with magnified insets showing colonies (bottom). The scale bar represents 200 μm. Colony forming efficiency in **G** was calculated and compared (right). Error bars indicate SEMs. **P=0.0015 (two-way ANOVA). **H**, Representative Ki67 immunostaining of liver metastases of the 13L-shRNA-1/2 organoids (left) and ratio of Ki67-positive tumor cells in liver metastases (right). Scale bars, 200 μm (top) and 100 μm (bottom). Each dot indicates individual mice. Error bars indicate SEMs. *p=0.0197, **P=0.0044 (two-way ANOVA). **Figure S5.** SOX2 is overexpressed in primary organoids. **A**, qRT-PCR analysis of SOX2 in primary organoid lines. Values were normalized to mean levels in control organoids. Error bars indicate SEMs. ****P < 0.0001 (one-way ANOVA). **B**, Western blot analysis of the SOX2 protein expression in primary organoid lines. α-tubulin was used as a loading control. **C**, Representative micrographs of colonies arising from the control, LV-Vector and LV-SOX2 primary organoid lines. The scale bar represents 200 μm. **D**-**F**, Colony forming efficiency in **C** was calculated and compared. Error bars indicate SEMs. ***P=0.0008, ****P < 0.0001 (one-way ANOVA). **G**, Proliferation of the control, LV-Vector and LV-SOX2 primary organoid lines were examined by CTG cell viability assays. Error bars indicate SEMs. ***P=0.0008, ****P < 0.0001 (one-way ANOVA). **H**, Representative micrographs of organoids in 3D invasion assay. Arrowheads, protrusive leading fronts. Micrographs of the paired organoids stained with phalloidin–F-actin. The scale bar represents 100 μm.

**Additional file 3.** Supplementary Materials and Methods.

## Data Availability

The datasets used and/or analyzed during the current study are available from the corresponding author on reasonable request.

## Abbreviations

CRC: Colorectal cancer
3D: Three-dimensional
MMP-2: Matrix metalloproteinase 2
qRT-PCR: Quantitative reverse transcription PCR
Dox: Doxycycline
CTG: CellTiter-Glo Luminescent
