# Supplementary material for: Modeling tumor development and metastasis using paired organoids derived from patients with colorectal cancer liver metastases
Source: J Hematol Oncol. 2020 Sep 3;13:119. doi: 10.1186/s13045-020-00957-4 (PMC7650218; doi:10.1186/s13045-020-00957-4)
Supplement: Supplementary file 2 — Additional file 2: Supplementary Figures. Figure S1. Paired organoids derived from primary and metastatic CRC recapitulate the histopathological structure of parental tumor. A and B, Organoids architecture resembles parental tumor epithelium. Representative bright-field images of organoids together with H&E staining of parental tumors and patient-derived organoids. The scale bar represents 100 μm. C and D, Representative IHC sections for the intestinal epithelial marker CDX2. The scale bar represents 100 μm. Figure S2. Organoids derived from liver metastatic lesions exhibited the most aggressive phenotypes with significant high tumorigenic and metastatic potential. A, Representative micrographs of organoids in 3D invasion assay. Tumor organoids showed the smooth and protrusive leading fronts, respectively. B, Micrographs of the paired organoids stained with phalloidin–F-actin (right). The scale bar represents 100 μm. C, qRT-PCR analysis of MMP-2 in paired organoid lines. Error bars indicate SEMs. *P=0.0207, **P =0.0083, ***P=0.0008 (one-way ANOVA; left), ***P=0.0006 (Unpaired t test; right). D, Western blot analysis of the MMP-2 protein expression in paired organoid lines. α-tubulin was used as a loading control. E, Representative IHC sections for Ki67 in human colorectal tumor tissues and paired organoid lines. The scale bar represents 100 μm. F, Representative IHC sections for Ki67 in organoid xenografts and organoids derived from xenografts. The scale bar represents 100 μm. G, Representative gross image, histopathology and Ki67 staining of whole liver from primary organoid xenografts. The scale bar of the whole liver represents 1cm. The black scale bar represents 100 μm. Figure S3. A, Representation of the up-regulated genes in 13L organoids. Error bars indicate SEMs. *P = 0.0283, ***P = 0.0004, ****P < 0.0001 (one-way ANOVA). B, qRT-PCR analysis of the up-regulated genes in 13L organoids. Values were normalized to mean levels in 13a organoids. Error bars indicat [file 13045_2020_957_MOESM2_ESM.docx]

# Modeling Tumor Development and Metastasis using Paired Organoids Derived from Patients with Colorectal Cancer Liver Metastases

He Li^1,2†^, Weixing Dai^3,4†^, Xi Xia^1†^, Renjie Wang^3,4†^, Jing Zhao^1,2^, Lingyu Han^3,4^, Shaobo Mo^3,4^, Wenqiang Xiang^3,4^, Lin Du^1^, Guangya Zhu^1^, Jingjing Xie^1^, Jun Yu^5^, Nan Liu^1^, Mingzhu Huang^4,6^*, Jidong Zhu^1,7^* and Guoxiang Cai^3,4^*

* Correspondence: mingzhuhuang0718@163.com, [zhujd@sioc.ac.cn](mailto:zhujd@sioc.ac.cn), [gxcai@fudan.edu.cn](mailto:gxcai@fudan.edu.cn)
^†^He Li, Weixing Dai, Xi Xa, and Renjie Wang contributed equally to this work.

1Interdisciplinary Research Center on Biology and Chemistry, Shanghai Institute of Organic Chemistry, Chinese Academy of Sciences, Shanghai, China. 2University of the Chinese Academy of Sciences, Beijing, China. 3Department of Colorectal Surgery, Fudan University Shanghai Cancer Center, Shanghai, China. 4Department of Oncology, Shanghai Medical College, Fudan University, Shanghai, China. 5Department of Surgery, Johns Hopkins University School of Medicine, Baltimore, Maryland, U.S.A. 6Department of Medical Oncology, Fudan University Shanghai Cancer Center, Shanghai, China. 7Center for Excellence in Molecular Synthesis, Shanghai Institute of Organic Chemistry, Chinese Academy of Sciences, Shanghai, China.

He Li ([lihe@sioc.ac.cn](mailto:lihe@sioc.ac.cn))

Weixing Dai ([daiweixing2015@163.com](mailto:daiweixing2015@163.com))

Xi Xia ([xiaxi@sioc.ac.cn](mailto:xiaxi@sioc.ac.cn))

Renjie Wang (wangbladejay@sina.com)

Jing Zhao ([zhaojing@sioc.ac.cn](mailto:zhaojing@sioc.ac.cn))

Lingyu Han ( [hanlingyu_ann@sina.cn](mailto:hanlingyu_ann@sina.cn))

Shaobo Mo ([shaobom@126.com](mailto:shaobom@126.com))

Wenqiang Xiang ([wqxiang2017@yeah.net](mailto:wqxiang2017@yeah.net))

Lin Du ([dulin@sioc.ac.cn](mailto:dulin@sioc.ac.cn))

Guangya Zhu ([zhugy@sioc.ac.cn](mailto:lihe@sioc.ac.cn))

Jingjing Xie ([xiejingjing@sioc.ac.cn](mailto:xiejingjing@sioc.ac.cn))

Jun Yu (jyu41@jhmi.edu)

Mingzhu Huang (mingzhuhuang0718@163.com)

Nan Liu ([liunan@sioc.ac.cn](mailto:liunan@sioc.ac.cn))

Jidong Zhu ([zhujd@sioc.ac.cn](mailto:zhujd@sioc.ac.cn))

Guoxiang Cai ([gxcai@fudan.edu.cn](mailto:gxcai@fudan.edu.cn))


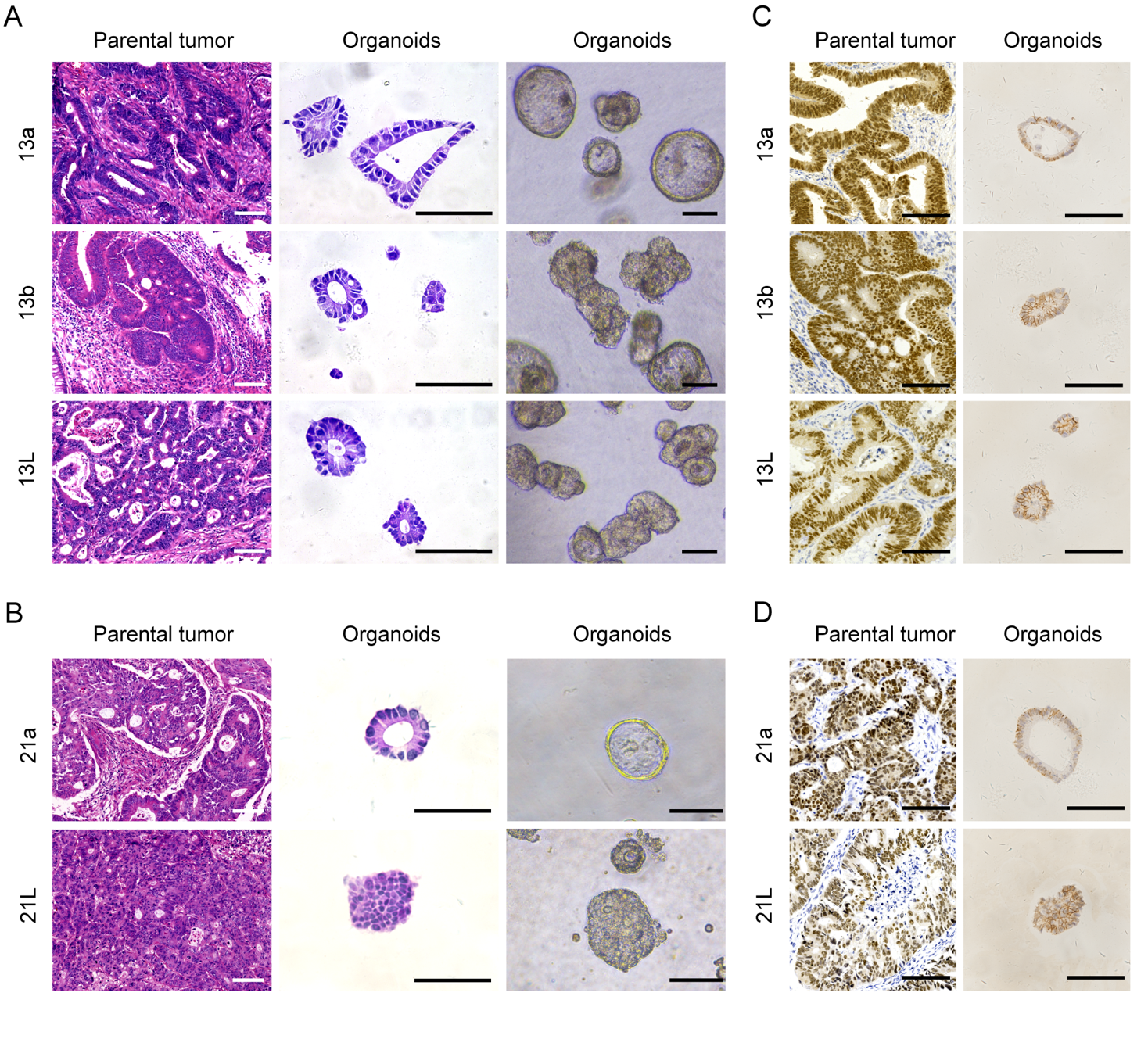


**Fig. S1** Paired organoids derived from primary and metastatic CRC recapitulate the histopathological structure of parental tumor. **A** and **B**, Organoids architecture resembles parental tumor epithelium. Representative bright-field images of organoids together with H&E staining of parental tumors and patient-derived organoids. The scale bar represents 100 μm. **C** and **D**, Representative IHC sections for the intestinal epithelial marker CDX2. The scale bar represents 100 μm.

**
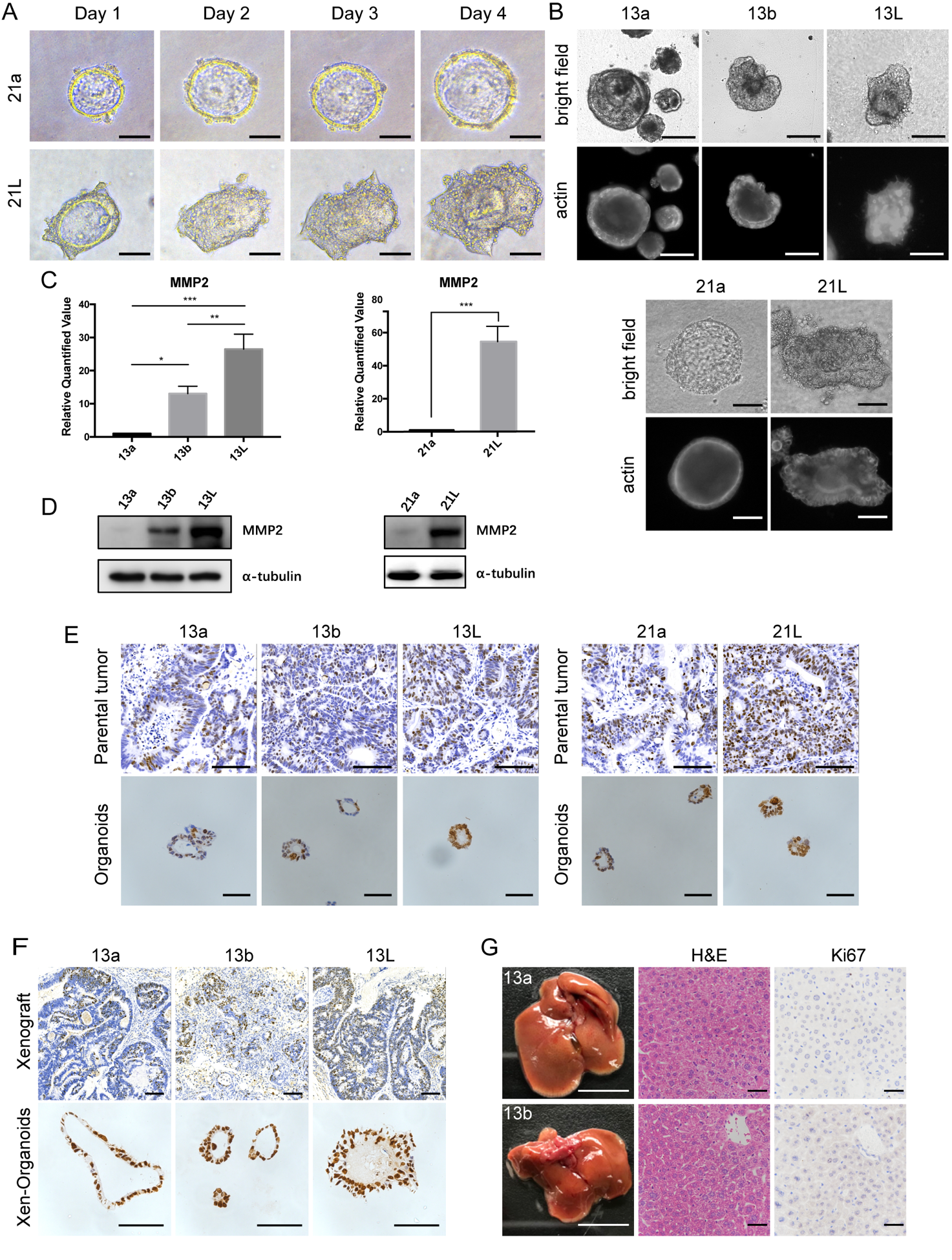
**

### Fig. S2 Organoids derived from liver metastatic lesions exhibited the most aggressive phenotypes with significant high tumorigenic and metastatic potential. A, Representative micrographs of organoids in 3D invasion assay. Tumor organoids showed the smooth and protrusive leading fronts, respectively. B, Micrographs of the paired organoids stained with phalloidin–F-actin (right). The scale bar represents 100 μm. C, qRT-PCR analysis of MMP-2 in paired organoid lines. Error bars indicate SEMs. *P=0.0207, **P =0.0083, ***P=0.0008 (one-way ANOVA; left), ***P=0.0006 (Unpaired t test; right). D, Western blot analysis of the MMP-2 protein expression in paired organoid lines. α-tubulin was used as a loading control. E, Representative IHC sections for Ki67 in human colorectal tumor tissues and paired organoid lines. The scale bar represents 100 μm. F, Representative IHC sections for Ki67 in organoid xenografts and organoids derived from xenografts. The scale bar represents 100 μm. G, Representative gross image, histopathology and Ki67 staining of whole liver from primary organoid xenografts. The scale bar of the whole liver represents 1cm. The black scale bar represents 100 μm.


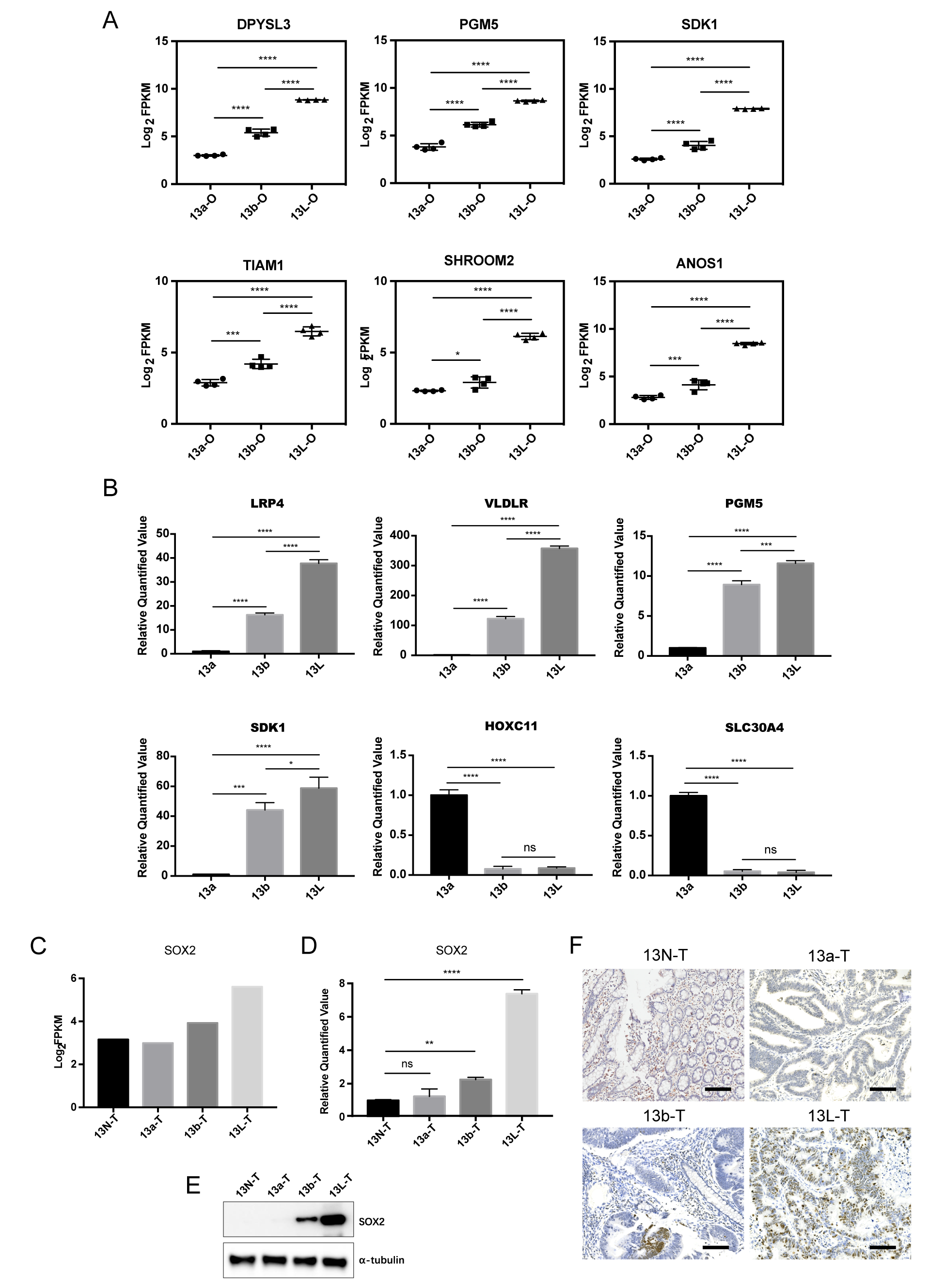


**Fig. S3** **A**, Representation of the up-regulated genes in 13L organoids. Error bars indicate SEMs. *P = 0.0283, ***P = 0.0004, ****P < 0.0001 (one-way ANOVA). **B**, qRT-PCR analysis of the up-regulated genes in 13L organoids. Values were normalized to mean levels in 13a organoids. Error bars indicate SEMs. *P=0.02, ***P=0.0008 ****P < 0.0001 (one-way ANOVA). **C**, RNA sequencing analysis of SOX2 in human colorectal cancer tissues and normal colon tissues. **D**, qRT-PCR analysis of SOX2 in tumor tissues and paired normal colon tissues. Values were normalized to mean levels in normal colon tissues. Error bars indicate SEMs. **P = 0.0018, ****P < 0.0001 (one-way ANOVA). **E**, Western blot analysis of the SOX2 protein expression in tumor tissues and paired normal colon tissues. α-tubulin was used as a loading control. **F**, Representative IHC sections for SOX2 in human colorectal tumor tissues and paired normal colon tissues. The scale bar represents 100 μm.


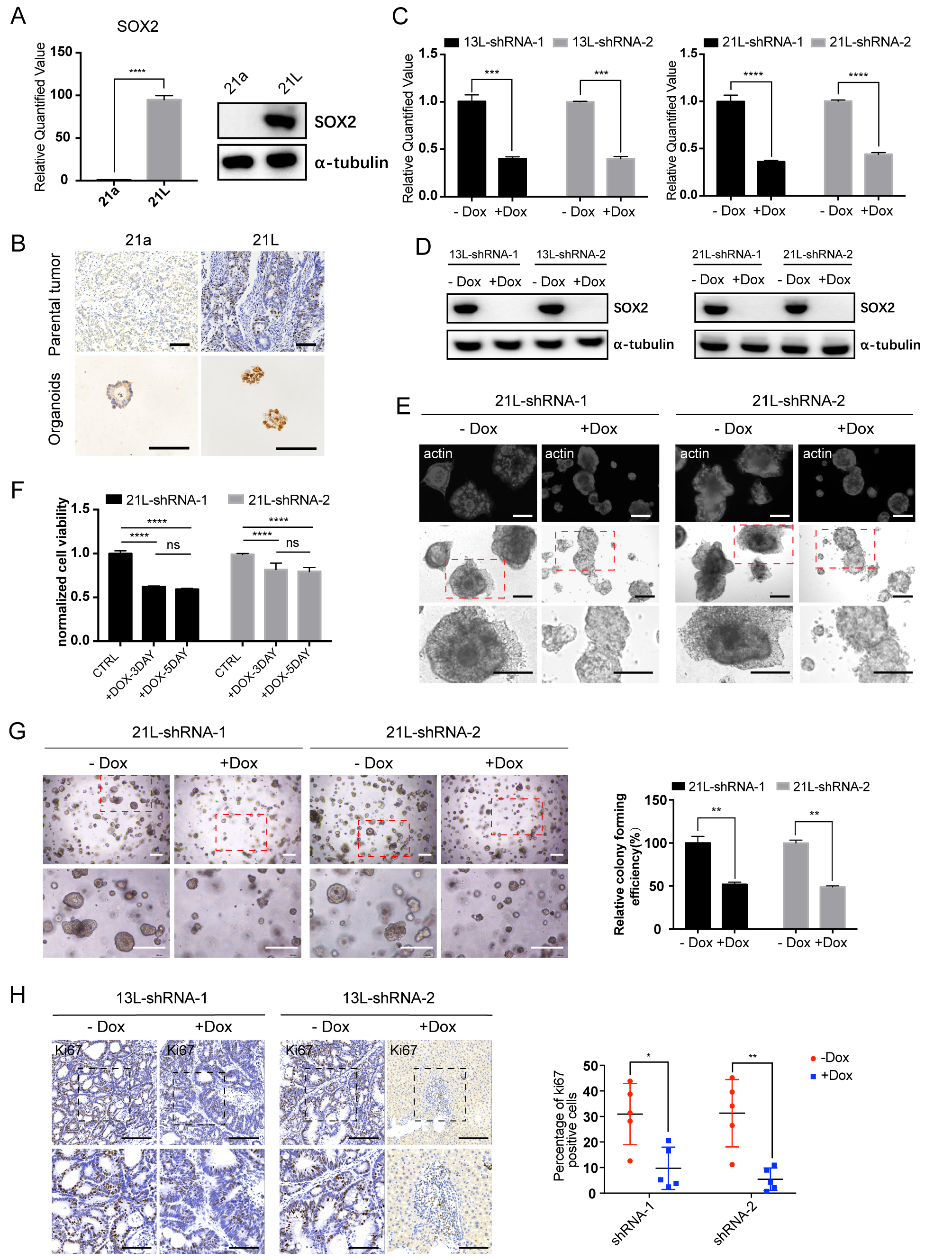


### Fig. S4 Silencing SOX2 in metastatic organoids attenuated cell invasion and proliferation and suppressed liver metastasis in vivo. A, qRT-PCR analysis of SOX2 in paired organoid lines (left). Values were normalized to mean levels in 21a organoids. Error bars indicate SEMs. ****P < 0.0001(Unpaired t test). Western blot analysis of the SOX2 protein expression in paired organoid lines. α-tubulin was used as a loading control (right). B, Representative IHC sections for SOX2 in human colorectal tumor tissues and organoids. The scale bar represents 100 μm. C, qRT-PCR analysis of SOX2 in the 13L-shRNA-1/2 (left) and 21L-shRNA-1/2 (right) organoids after 3 days of Dox-induction. The level of SOX2 was compared to that in the untreated sample. Error bars indicate SEMs. ***P=0.0001, ****P < 0.0001 (two-way ANOVA). D, Western blot analysis of the expression of SOX2 in the 13L-shRNA-1/2 and 21L-shRNA-1/2 organoids after 3 days of Dox-induction. α-tubulin was used as a loading control. E, Representative micrographs of Dox-transduced the 21L-shRNA-1/2 organoids after 5 days and stained with phalloidin–F-actin. The scale bar represents 100 μm. F, Proliferation of the 21L-shRNA-1/2 organoids were examined by CTG cell viability assays following 3- or 5-days growth in the presence or absence of Dox. Error bars indicate SEMs. **p=0.0019, ****P < 0.0001 (two-way ANOVA). G, Representative micrographs of colonies arising from the 21L-shRNA-1/2 organoids (top), with magnified insets showing colonies (bottom). The scale bar represents 200 μm. Colony forming efficiency in G was calculated and compared (right). Error bars indicate SEMs. **P=0.0015 (two-way ANOVA). H, Representative Ki67 immunostaining of liver metastases of the 13L-shRNA-1/2 organoids (left) and ratio of Ki67-positive tumor cells in liver metastases (right). Scale bars, 200 μm (top) and 100 μm (bottom). Each dot indicates individual mice. Error bars indicate SEMs. *p=0.0197, **P=0.0044 (two-way ANOVA).


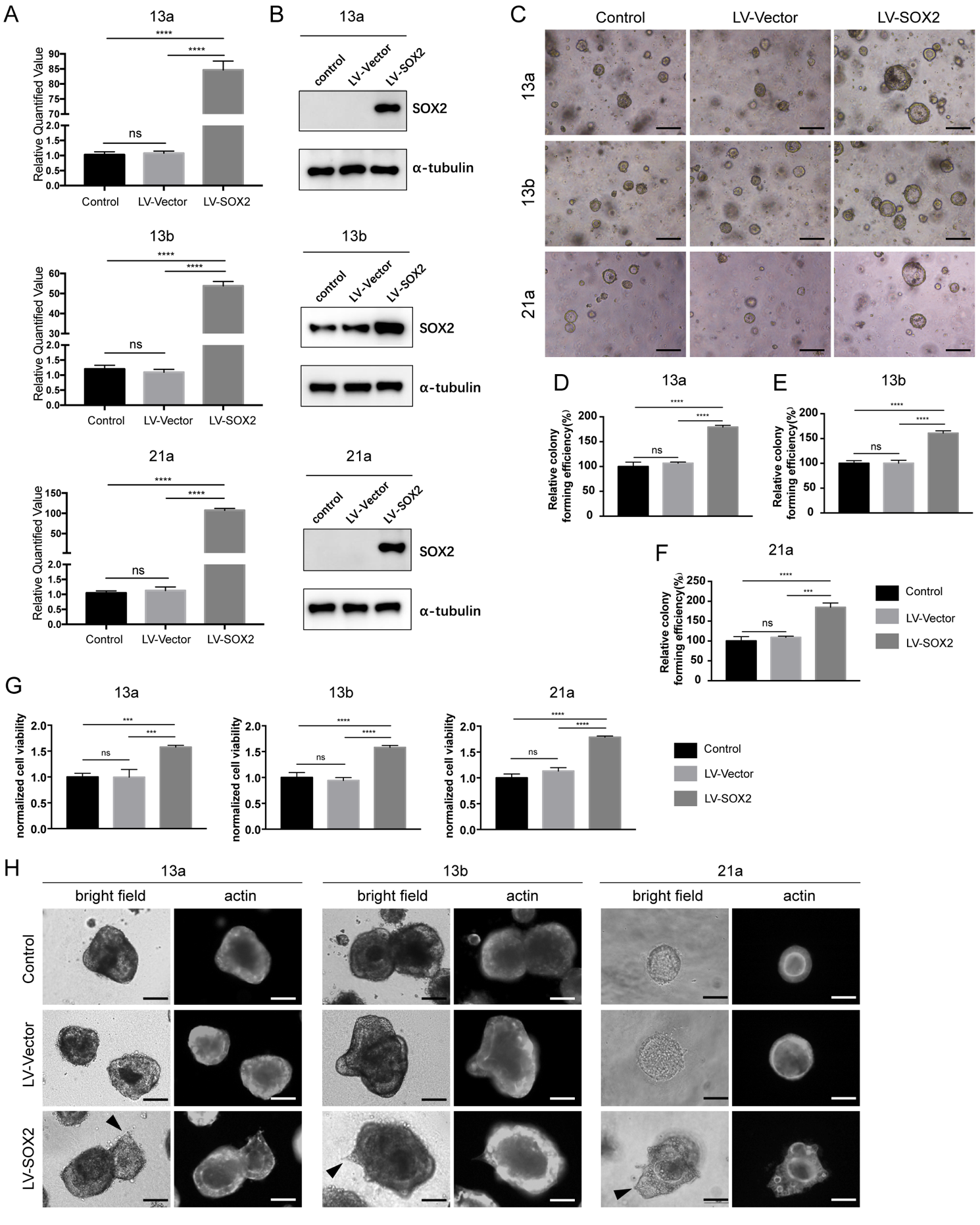


**Fig. S5** SOX2 is overexpressed in primary organoids. **A**, qRT-PCR analysis of SOX2 in primary organoid lines. Values were normalized to mean levels in control organoids. Error bars indicate SEMs. ****P < 0.0001 (one-way ANOVA). **B**, Western blot analysis of the SOX2 protein expression in primary organoid lines. α-tubulin was used as a loading control. **C**, Representative micrographs of colonies arising from the control, LV-Vector and LV-SOX2 primary organoid lines. The scale bar represents 200 μm. **D**-**F**, Colony forming efficiency in **C** was calculated and compared. Error bars indicate SEMs. ***P=0.0008, ****P < 0.0001 (one-way ANOVA). **G**, Proliferation of the control, LV-Vector and LV-SOX2 primary organoid lines were examined by CTG cell viability assays. Error bars indicate SEMs. ***P=0.0008, ****P < 0.0001 (one-way ANOVA). **H**, Representative micrographs of organoids in 3D invasion assay. Arrowheads, protrusive leading fronts. Micrographs of the paired organoids stained with phalloidin–F-actin. The scale bar represents 100 μm.
