# Supplementary material for: Modeling tumor development and metastasis using paired organoids derived from patients with colorectal cancer liver metastases
Source: J Hematol Oncol. 2020 Sep 3;13:119. doi: 10.1186/s13045-020-00957-4 (PMC7650218; doi:10.1186/s13045-020-00957-4)
Supplement: Supplementary file 3 — Additional file 3. Supplementary Materials and Methods. [file 13045_2020_957_MOESM3_ESM.docx]

## Supplementary Materials and Methods

### Human Tissues

All samples used for organoid establishment were obtained from Fudan University Shanghai Cancer Center Hospital (Shanghai, China). Written informed consent was obtained prior to acquisition of tissue from all patients. The studies were conducted in accordance with recognized ethical guidelines (Declaration of Helsinki) and approved by the Institutional Review Board of Fudan University Shanghai Cancer Center. Healthy and neoplastic colonic tissues were obtained from either endoscopic biopsy or surgically resected specimens. The samples were kept in ice-cold PBS until processing.

### Tissue dissociation and organoid culture

The patient-derived organoids were prepared from large intestine tumors, as described previously [1, 2], with several modifications. In short, tumors were cut into pieces and two parts were processed for immunohistochemistry and RNA isolation. The remainder was cut into smaller pieces and incubated in digestion buffer Advanced DMEM/F12 (Thermo Fisher Scientific, USA) medium with 2.5% fetal bovine serum, 1% penicillin/streptomycin (Invitrogen, USA), 75 U/mL collagenase type IX (Sigma-Aldrich, USA) ,125 g/mL dispase type II (Invitrogen, USA) for 30 minutes at 37^o^C while shaking. After incubation, Basal culture medium (Advanced DMEM/F12 supplemented with penicillin/streptomycin, 10 mM HEPES (Invitrogen, USA) and 2 mM GlutaMAX (Invitrogen, USA) was added and the mixture was put over a 100 μm cell strainer (Falcon, USA) to remove large fragments. Cells were subsequently spun at 1,000 rpm for 3 min. The pellet was resuspended in basal culture medium and spun again at 1,000 rpm. The procedure was repeated twice to remove debris and collagenase. The tumor material was resuspended in Basement Membrane Extract (BME; Cultrex RGF Basement Membrane Extract, Type 2; R&D Systems, USA), and dispensed into 24-well culture plates (40ul BME/well). The BME was then solidified by a 20 minute incubation in a 37 ^o^C and 5% CO_2_ cell culture incubator, and overlaid with 500 µl of complete human organoid media; The composition of CRC culture medium is: Basal culture medium supplemented with 20% R-spondin-1 conditioned medium [3], 100 ng/ml mouse recombinant Noggin (PeproTech, USA), 1X B27 (Invitrogen, USA), 1.25 mM n-Acetyl Cysteine (Sigma-Aldrich, USA), 10 mM Nicotinamide (Sigma-Aldrich, USA), 50 ng/ml EGF (PeproTech, USA), 10 nM Gastrin(Sigma-Aldrich, USA), 500 nM A83-01 (MedChemExpress, USA), 5μM SB202190 (MedChemExpress, USA) , 10 nM Prostaglandine E2 (MedChemExpress, USA) and 100 µg/ml Primocin (Invitrogen, USA). complete media was subsequently refreshed every two days.

For passaging, BME was broken up by pipetting and organoids were collected in a tube. The organoids were centrifuged at 1,000 rpm for 3 min and the medium removed. 1x TrypLe Express (Thermo Fisher Scientific, USA) was added and the organoids were incubated at 37 ^o^C for approximately 5 min. Organoids were then dissociated to small cell clusters by applying mechanical force (pipetting), washed with HBSS (Thermo Fisher Scientific, USA), pelleted (1,200rpm, 5 min, 4 ^o^C), resuspended in BME, and re-seeded at an appropriate ratio. Mycoplasma testing was done by nested PCR. Organoids were biobanked in FBS (Thermo Fisher Scientific, USA), containing 10% DMSO (SigmaAldrich, USA).

### Histology and immunohistochemistry

Organoids were harvested and fixed with 4% paraformaldehyde (Sigma-Aldrich, USA) overnight. Following fixation, Organoids were washed and resuspended in 200 µl of warm (~45 ^o^C) agarose 1% (in H_2_O). The agarose pellet was left to set, dehydrated using ethanol, and embedded in paraffin using a standard histological protocol. Organoid and tissue H&E staining were conducted following a standard staining protocol. Immunohistochemistry was then continued following the manufacturer’s instructions. The following primary antibodies were used for immunohistochemical staining: Ki67 (#9449, Cell Signaling, USA), 1:250; CDX2 (#12306, Cell Signaling, USA), 1:2000; SOX2 (11064-1-AP, Proteintech, USA), 1:200. Imaging of stained organoids was performed under a Nikon ECLIPSE Ni-U upright microscope (Nikon, Japan).

### 3D invasion assay of organoids

Harvest organoids and resuspend in 3D Invasion Matrix (3500-096-03, Trevigen, USA). Aliquot 10 µl of cell suspension per well of the standard 96-well cell culture plates (Corning, USA). Transfer plate to in a 37 ^o^C and 5% CO_2_ cell culture incubator for one hour to promote gel formation of the Invasion Matrix. After one hour, add 100 µl of cell culture medium. Incubate at a 37 ^o^C and 5% CO_2_ cell culture incubator for 3 to 6 days and brightfield imaging of organoids in each well was performed every 24 hours with ECLIPSE TS100 inverted microscope (Nikon, Japan). Adjust the lighting and focus to provide the most contrast between the 3D structure and background. The assay may be conducted longer than 6 days if desired; the endpoint is usually when the structure size begins to exceed the field of analysis or the organoids begin to expire.

For F-actin staining of organoids cultured in 3D Invasion Matrix, Matrix gels were fixed in 4% paraformaldehyde for 10 min, washed twice in PBS for 10 min, permeabilized with 0.2% Triton X-100 (Sigma-Aldrich, USA) in PBS for 30 min, and washed twice in PBS for 10 min. Phalloidin (40734ES75, Yeasen Biotech Co., China) was added at 1:200 dilution to stain F-actin positive cell membranes. For these experiments, imaging was performed with Leica DMi8 Inverted microscope (Leica, Germany).

### Organoid xenograft assays

All animal procedures were performed under guidelines approved by the Institutional Animal Care and Use Committee of the Shanghai Institute of Organic Chemistry, Chinese Academy of Sciences. All mice were obtained from the Shanghai Experimental Animal Center (Shanghai, China). The organoids were harvested using the passaging procedure described above and resuspended in 50% Matrigel (Corning, USA)/50% organoid culture media at a concentration of 10^7^ cells/ml. A total 200 µl (2 ×10^6^ cells) of organoid suspension was injected subcutaneously into BALB/c nude mice (6-8 weeks old, male). The tumor volumes were measured every 4 day and calculated by using the standard formula: length × width^2^/2. After 60 days, mice were euthanized. In some xenografts, fresh tumor samples were used for the establishment of xenograft-derived organoids according to the methods described above. Some samples were then 4% paraformaldehyde-fixed for subsequent immunohistochemical analysis. Each organoid line was transplanted into five independent BALB/c nude mice. For hepatic metastasis assay by splenic organoid injection. The organoids were prepared with the same procedure described above. A total 10^6^ cells of Matrigel-organoid suspension were injected into the spleens of NOD/scid (NOD) mice (6-8 weeks old, male). Two months after transplantation, the livers were isolated. Each organoid line was transplanted into five independent NOD mice. Immunohistochemical analysis was performed on some samples.

### RNA sequencing

Total RNA was extracted from organoids 4-6 days after passaging and organoid-matching tumors using an AllPrep DNA/RNA Mini Kit (Qiagen, Germany). A total amount of 1 µg RNA per sample was used for sequencing library preparation. The quality of each sample was measured using the RNA Nano 6000 Assay Kit of the Bioanalyzer 2100 system (Agilent Technologies, USA). PolyA-tailed RNAs were selected by NEB Next Poly(A) mRNA Magnetic Isolation Module (NEB, USA), followed by the library prep using NEB Next Ultra RNA library Prep Kit for Illumina according to manufacturer’s instruction (NEB, USA). The quantification was performed by qRT-PCR with a reference to a standard library. The libraries were pooled together in equimolar amounts to a final 2 nM concentration. The normalized libraries were denatured with 0.1 M NaOH (Sigma-Aldrich, USA). Pooled libraries were sequenced on the illumina Xten platforms with PE150 (illumina, USA). Sequence quality was examined using the FastQC software. Sequencing reads were mapped to the reference genome Rnor_6.0 from ensembl with STAR-2.6.0 [4] by default parameter. HTSeq (v0.6.0) was used to count the reads numbers mapped to each gene. And then Fragments per Kilobase Million (FPKM) of each gene was calculated based on the length of the gene and reads count mapped to this gene. The raw and normalized data files are deposited in Gene Expression Omnibus (GEO) with series entry number GSE148918.

### RNA-seq Data Analysis

Differentially expressed genes in paired organoids and tissue samples were identified using the DESeq2 package [5]. Genes with an adjusted P values of < 0.05 found by DESeq2 were assigned as differentially expressed. Principal component analysis performed using the prcomp function implemented in the R software (https://www.r-project.org). Gene Set Enrichment Analysis was applied to identify significantly changed pathways [6]. P values of < 0.05 were considered significantly enriched by differential expressed genes. Heatmaps and hierarchal clustering were performed using Morpheus available from (https://software.broadinstitute.org/morpheus/). P values of < 0.05 were considered statistically significant.

### Quantitative reverse transcription-PCR

Total RNA from organoids and organoid-matching tumors was extracted using an AllPrep DNA/RNA Mini Kit (Qiagen, Germany). cDNA was synthesized with purified RNA using Hifair Ⅱ 1st Strand cDNA Synthesis SuperMix for qPCR (gDNA digester plus) (Yeasen Biotech Co., China). Amplification was performed using primers with TB Green Premix Ex Taq (TAKARA, Japan) using IQ5 Realtime-PCR instrument (Life Technologies, USA). Quantitative reverse transcription-PCR (qRT-PCR) assays were set up in triplicate. All protocols were performed according to the manufactures’ instructions. Results were analyzed with the ΔΔCT method, using GAPDH as internal reference gene. The primer sequences for genes are as follows：

SOX2:

Forward Primer-1: GCCGAGTGGAAACTTTTGTCG

Reverse Primer-1: GGCAGCGTGTACTTATCCTTCT

Forward Primer-2: TGGACAGTTACGCGCACAT

Reverse Primer-2: CGAGTAGGACATGCTGTAGGT

GAPDH:

Forward Primer-1: GGAGCGAGATCCCTCCAAAAT

Reverse Primer-1: GGCTGTTGTCATACTTCTCATGG

Forward Primer-2: ACAACTTTGGTATCGTGGAAGG

Reverse Primer-2: GCCATCACGCCACAGTTTC

MMP2:

Forward Primer-1: TACAGGATCATTGGCTACACACC

Reverse Primer-1: GGTCACATCGCTCCAGACT

Forward Primer-2: CCCACTGCGGTTTTCTCGAAT

Reverse Primer-2: CAAAGGGGTATCCATCGCCAT

### Western blot analysis

For protein preparation from fresh tissues, the tissues were homogenized with homogenizers and lysed in RIPA lysis buffer (Sigma-Aldrich, USA) containing a protease inhibitor cocktail (Selleck Chemicals, USA). For protein preparation from the organoids, the organoids were harvested and lysed in RIPA buffer (Sigma-Aldrich, USA) supplemented with protease inhibitor cocktail (Selleck Chemicals, USA). Protein concentration in each sample was quantified using BCA protein assay kits (Thermo Fisher Scientific, USA). The samples of the same amount of protein were loaded and resolved by SDS–PAGE, transferred to PVDF membrane (GE Healthcare Life Sciences, USA). Primary antibodies were diluted at 1:1,000 in TBS-T (TBS + 0.2% Tween) containing 5% BSA, and staining was conducted overnight at 4 ^o^C. Primary antibodies used in the study included rabbit SOX2 (11064-1-AP, Proteintech, USA), rabbit MMP-2 (#40994, Cell Signaling, USA), rabbit α-tubulin (#2125, Cell Signaling, USA). Secondary antibodies (anti-rabbit IgG HRP-linked, Cell Signaling, USA) were diluted 1:10,000 in TBS-T containing 5% skimmed milk. Protein bands were developed with an enhanced chemiluminescence substrate (Beyotime Biotechnology, China) and captured with GE-AI-600 imaging system (GE Healthcare Life Sciences, USA).

### Lentiviral knockdown of SOX2 in organoids and xenograft assays

For inducible knockdown of SOX2, organoids were engineered for Dox-inducible expression of an shRNA using a pLKO-Tet-On vector. shRNA sequences from The RNAi Consortium collection (MISSION® shRNA, Sigma, www.sigmaaldrich.com), which utilizes constitutive pLKO lentiviral vector, can be subcloned into the Dox-inducible expression of an shRNA using a pLKO-Tet-On vector between the AgeI and EcoRI restriction sites. For lentiviral packaging, Lentivirus was produced in 293T cells by transfecting plasmids and packaging plasmids with PolyJet In Vitro DNA transfection reagent (SignaGen, USA) according to the protocol described by the manufacturer. Lentivirus infection of organoids, as previously described [7]. Briefly, organoids were dissociated into single cells and pelleted by centrifugation. Pellets were resuspended in complete organoid media and viral supernatant at a 1:1 ratio (500 µl each), with the addition of 8 µg/ml polybrene. The cell solution was transferred in a well of a 12-well plate and left overnight in a 37 ^o^C and 5% CO_2_ cell culture incubator. The following morning cells were harvested using PBS-EDTA 1mM containing 1x TrypLe, pelleted, seeded in BME, and overlaid with complete medium. Two days post infection 2 µg/mL of puromycin (Sigma-Aldrich, USA) was added to the complete organoid media, and selection of infected cells was done for a period of 1-2 weeks.

Two shRNAs targeting human SOX2 designed as:

SOX2 shRNA1 (Clone ID: NM_003106.2-1060s21c1; Sigma-Aldrich, USA) CCGGCAGCTCGCAGACCTACATGAACTCGAGTTCATGTAGGTCTGCGAGCTGTTTTTG

SOX2 shRNA2 (Clone ID: NM_003106.2-780s21c1; Sigma-Aldrich, USA)

CCGGCGCTCATGAAGAAGGATAAGTCTCGAGACTTATCCTTCTTCATGAGCGTTTTTG

For induced SOX2 knockdown experiments, each shRNA-organoid line was transplanted into five independent NOD mice. i-KdSOX2 organoids were prepared as described above. After the spleen injection, NOD mice were randomized to control (Dox-untreated) and experimental (Dox-treated) groups. Dox-treatment for knockdown of SOX2 was accomplished by addition of Dox (2 mg/ml, Sigma-Aldrich, USA) to drinking water that contained 5% sucrose. Untreated mice were provided with 5% sucrose drinking water as a control. Two months after transplantation, mice were euthanized, the livers were isolated. Tumor tissue samples were prepared for subsequent IHC. The mean Ki67-labeling indices were calculated as the number of Ki67-positive cells per total number of tumor cells by counting 5 independent microscopic fields (200×) for each mouse.

**Organoids engineering for SOX2** **overexpression**

The human SOX2 CDS region was cloned from human genomic DNA and was confirmed by sequencing. Then, the PCR product was subcloned into pLV-EF1a-IRES. The pLV-EF1a-IRES was used as the control plasmid. For lentiviral packaging and lentivirus infection as described above. Two days post infection 2 µg/mL of puromycin (Sigma-Aldrich, USA) was added to the complete organoid media, and selection of infected cells was done for a period of 1-2 weeks.

### Colony formation assay

Organoids were harvested and dissociated into single cells. Cell pellets were resuspended in PBS, cells were counted with the Countess automated cell counter (Thermo Fisher Scientific, USA). Subsequently, cells were resuspended in BME (2 × 10^6^ cells/ml) and seeded in standard 96-well cell culture plates (Corning, USA) in triplicate. The cells were cultured with complete human organoid media, supplemented with vehicle controls or 1 µM of Dox. Complete media was refreshed every three days. For seven days. The number of colonies formed in each well was assessed using a Nikon ECLIPSE TS100 bright field inverted microscope (Nikon, Japan). Analyze images using ImageJ software to measure colony forming efficiency for each sample.

### Organoid Viability Assay

Organoids were collected 2-3 days after passaging and passed through a 40μm cell strainer (Falcon, USA) to eliminate large organoids. Subsequently, organoids were resuspended in 5% BME /organoid culture medium (15,000-20,000 organoids/ml) and dispensed into ultralow-attachment 96-well plates (Corning, USA) in triplicate. The organoids were treated with vehicle controls or 1 µM of Dox. Cell viability was assayed using CellTiter-Glo (Promega, USA) according to the manufacturer’s instructions following 3 or 5 days of Dox incubation, and results were normalized to vehicle controls.

### Statistical analysis

All the experiments were performed at least in triplicates, and the values were presented as means ± SD with sample size. Figure legends describe the statistical test used for each experiment and other parameters associated with the representation of the data. The difference was considered statistically significant if a P value was less than 0.05. For the studies in vivo, animal randomization was performed before all the experiments.

## Supplemental References

1. van de Wetering M, Francies HE, Francis JM, Bounova G, Iorio F, Pronk A, et al. Prospective derivation of a living organoid biobank of colorectal cancer patients. *Cell*. 2015;161(4):933–945.
2. Sato T, Stange DE, Ferrante M, Vries RGJ, Van Es JH, Van den Brink S, et al. Long-term expansion of epithelial organoids from human colon, adenoma, adenocarcinoma, and Barrett's epithelium. *Gastroenterology*. 2011;141(5):1762–1772.
3. Ootani A, Li X, Sangiorgi E, Ho QT, Ueno H, Toda S, et al. Sustained in vitro intestinal epithelial culture within a Wnt-dependent stem cell niche. *Nat Med*. 2009;15(6):701–706.
4. Dobin A, Davis CA, Schlesinger F, Drenkow J, Zaleski C, Jha S, et al. STAR: ultrafast universal RNA-seq aligner. *Bioinformatics*. 2013;29(1):15–21.
5. Love MI, Huber W, Anders S. Moderated estimation of fold change and dispersion for RNA-seq data with DESeq2. *Genome Biol*. 2014;15(12):550.
6. Subramanian A, Tamayo P, Mootha VK, Mukherjee S, Ebert BL, Gillette MA, et al. Gene set enrichment analysis: a knowledge-based approach for interpreting genome-wide expression profiles. *Proc Natl Acad Sci U S A*. 2005;102(43):15545–15550.
7. Vlachogiannis G, Hedayat S, Vatsiou A, Jamin Y, Fernández-Mateos J, Khan K, et al. Patient-derived organoids model treatment response of metastatic gastrointestinal cancers. *Science* 2018;359: 920–926.
